# Unusual manifestation of COVID‐19‐associated pulmonary aspergillosis: A case report

**DOI:** 10.1002/ccr3.6188

**Published:** 2022-08-11

**Authors:** Maryam Albaji, Mohammad Reza Fattahi, Arad Iranmehr

**Affiliations:** ^1^ Department of Pulmonary Disease Sina Hospital, Tehran University of Medical Sciences Tehran Iran; ^2^ Department of Radiology Sina Hospital, Tehran University of Medical Sciences Tehran Iran; ^3^ Research Center for Clinical Virology Tehran University of Medical Sciences Tehran Iran; ^4^ Neurological Surgery Department, Imam Khomeini Hospital Complex Tehran University of Medical Sciences Tehran Iran

**Keywords:** CAPA, cavitary, neurologic, superinfection

## Abstract

A young man, a recent coronavirus patient, was readmitted with hypoesthesia and dysarthria following a rapid deterioration of respiratory symptoms. The brain and lung CT scans revealed ischemia and cavitary lung lesions. Clinical suspicion for aspergillus leads to prompt treatment, confirmed by biopsy. Neurologic and pulmonary symptoms resolved ultimately.

## Graphical abstract

This case report highlights the potentially effective and on‐time diagnosis and treatment of the aspergillus involvement in a non‐immunocompromised patient with COVID‐19 admitted with a neurological ischemic symptom.

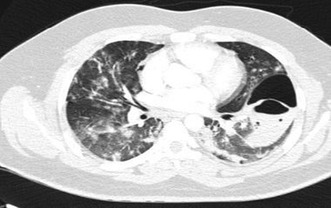

## 1 INTRODUCTION

Since December 2019, an outbreak of coronavirus pneumonia caused by SARS‐COV‐2 started in Wuhan, China,^1^ and later spread worldwide. Severe viral pneumonia‐causing hospitalization and respiratory failure is an essential feature of this disease.^2^ Invasive aspergillosis infections have been reported in severe respiratory syndrome‐coronavirus (SARS‐CoV) in 2003 and Middle East Respiratory Syndrome‐coronavirus.^3^, ^4^ In critically ill patients of influenza, the influenza‐associated pulmonary aspergillosis (IAPA) is a risk factor for morbidity.^5^, ^6^ Several studies from Wuhan,^7^, ^8^ Belgium,^9^ France,^10^ Netherland,^11^ and Germany^12^ have reported COVID‐19 associated pulmonary aspergillosis (CAPA) in critically ill patients and mainly on mechanical ventilation. Corticosteroid use in the treatment of COVID‐19 patients may contribute to CAPA risk. This report presents a young man with neurologic presentation and cavitary lung lesions with a history of recent hospitalization for moderate COVID‐19 pneumonia.

## 2 CLINICAL CASE

A 30‐year‐old male patient was admitted to the emergency room with a chief complaint of hypoesthesia in both lower limbs, right hand, and dysarthria since the day before. He had no history of loss of consciousness or head trauma, diplopia, dysphagia, facial weakness, fecal or urinary incontinence, dysarthria, nausea or vomiting, articular pain or swelling, oral ulcer, or photosensitivity nasal discharge. However, he was complaining of headaches, fever, and vertigo. The patient also mentioned exertional dyspnea since getting infected with COVID‐19, which led to hospitalization 20 days ago. The patient's past medical history had no significant reporting except for the recent admission for COVID‐19 infection, which was treated with Remdesivir and Dexamethasone for 5 days in another healthcare center. His habitual history was positive for hookah, but no further history of smoking or alcohol use was mentioned. There were construction works in the patient's neighborhood recently. He noted that his mother has a history of hypertension. The patient's signs improved since their onset but did not resolve completely. In the physical examination, the patient was slightly ill but had no respiratory distress; blood pressure was 110/80 mmHg, the pulse rate was 80 b/min, respiratory rate of 18/min, the temperature of 36.8°C, and O_2_ saturation was 96%.

**FIGURE 1:**
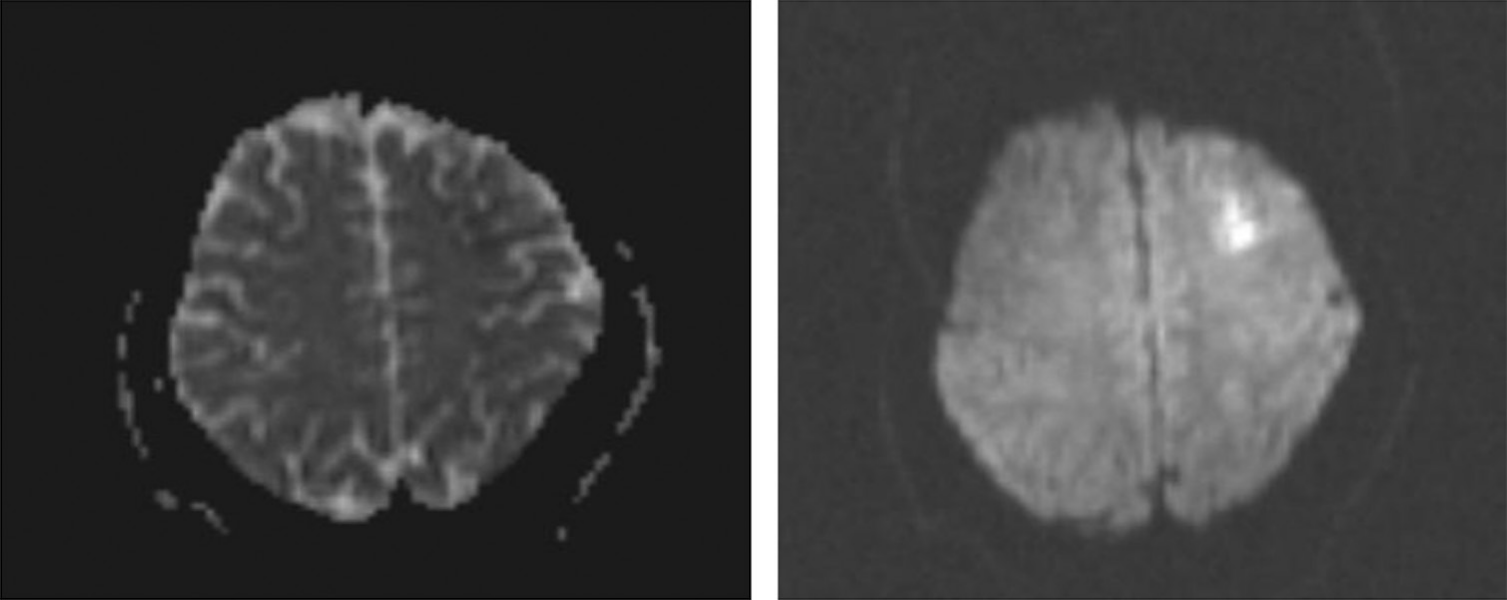
Brain MRI revealed abnormal signal intensity in the left frontal region in T2/FLAIR, which shows restriction in DWI image in favor of acute infarction in left MCA territory

Moreover, muscle forces and the other neurologic examinations were normal. Lung and brain CT scans were done and had no obvious abnormality. Brain MRI was performed, and there was an abnormal signal intensity in the left frontal region in T2/FLAIR, which indicates a restriction in diffusion‐weighted imaging (DWI) in favor of acute infarction in the left middle cerebral artery (MCA) territory (Figure 1). The bilateral carotid Doppler ultrasound revealed low resistance arterial flow without evidence of significant narrowing or dissection. Initial laboratory data are shown in Table 1.

**TABLE 1:** Laboratory findings of the patient

Laboratory test	Result	Reference
WBC count	13,200/HPF	4000–11,000/HPF
CRP	8.2 mg/L	0–10
ESR	36 mm/h	up to 18
LDH	509 U/L	Up to 480 U/L
D‐Dimer	292 ng/ml	Up to 500 ng/ml
Troponin I. Quantitative	139.2 ng/ml	Up to 26 ng/ml
COVID‐19/PCR Qualitative	Not detected	Negative

**FIGURE 2:**
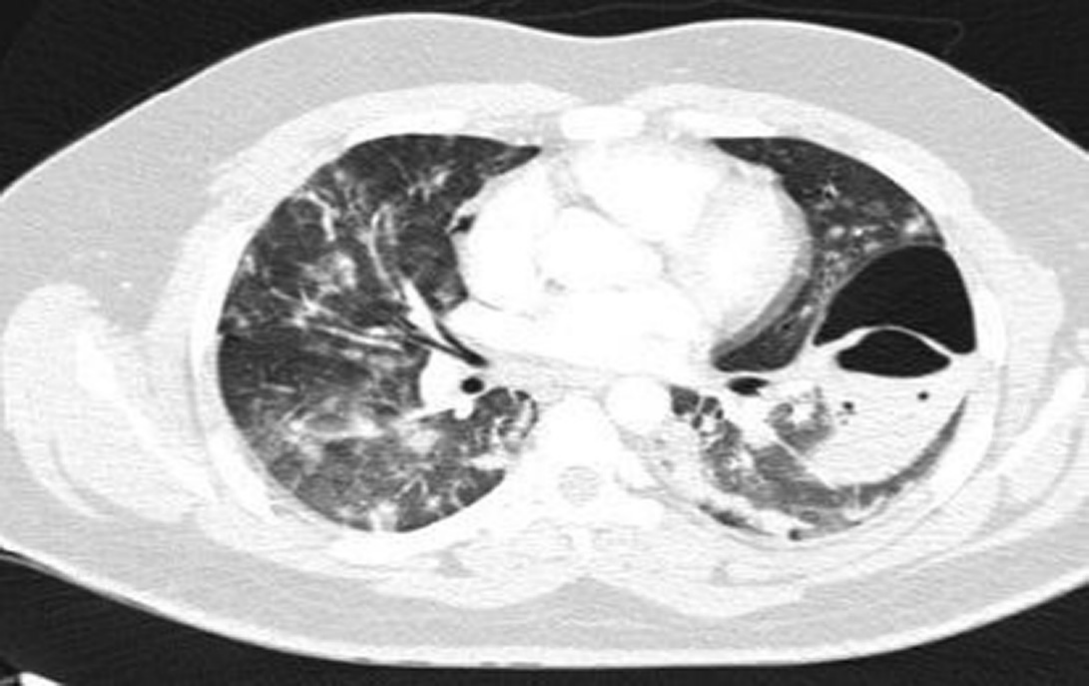
Chest CT scan in the ER on the second day of the presentation

Neurologic consult recommended treatment with Aspirin (80 mg oral intake daily), Clopidogrel (75 mg oral intake daily), and Atorvastatin (20 mg oral intake daily). On the day after admission, the patient had dyspnea, and the level of O_2_ saturation dropped to 77% with rose to 95% with oxygen therapy, and he developed tachycardia (PR = 120b/min). Lung CT angiography to rule out pulmonary thromboembolism (PTE) was performed. There was no evidence of thrombosis in the main and segmental branches of the pulmonary artery. There was evidence of previous COVID‐19 infection in lung parenchyma and nodular opacities with some cavitation suggesting superimposed infections or possibly due to septic emboli (Figure 2). Broad‐spectrum antibiotics were initiated, and blood cultures were sampled. Transthoracic echocardiography (TTE) was done, which had a poor view due to the patient's obesity and tachypnea; then, transesophageal echocardiography (TEE) was performed, which reported normal RA size with a moderate, fluffy, network‐shaped mobile mass in the right atrial (RA); however, the operator could not determine the lesion was a pathologic or not. The other findings were normal and had no evidence of RV strain, vegetation, or significant valve abnormality, and cardiac MRI was suggested for better evaluation. The patient's exertional dyspnea was aggravated, resulting in immobility; therefore, the patient needed urinary catheterization. For reevaluation, we repeated pulmonary CT angiography. The cavitary lesions progressed in size and distribution with a dominant cavity sized 107, 70, and 50 mm in the superior segment of the left lower lobe with internal fluid level septation, suggesting fungal infections. An infectious disease specialist consults recommended antifungal therapy with intravenous voriconazole (200 mg every 12 h) was started. Sputum examination for fungal and bacterial infections was negative. Bronchoscopy and bronchoalveolar lavage were performed, and the smear and culture for alveolar lavage were negative for bacterial and fungal infection. The patient had an episode of hematuria; urine analysis revealed microscopic hematuria without dysmorphic RBC and pyuria.

**FIGURE 3:**
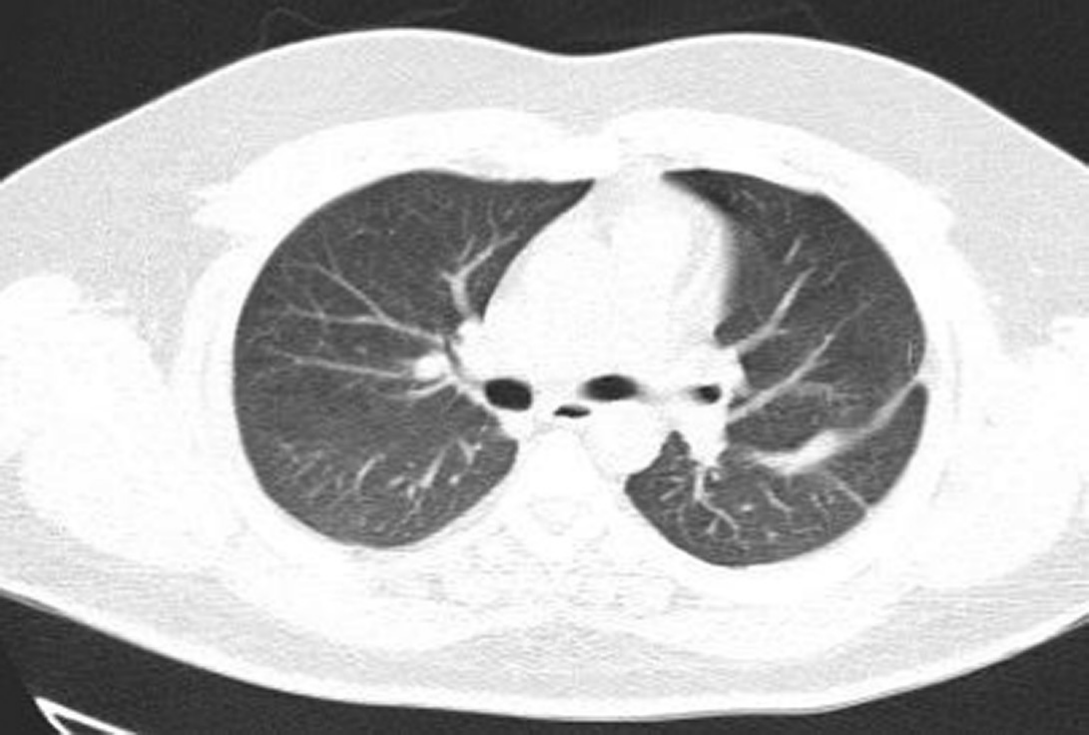
Chest CT scan after complete treatment

Evaluation for vasculitis because of hematuria, cavitary lung lesions, and neurologic presentation of the disease by testing for ANA, Anti ds DNA, RF, Anti CCP Ab, P‐ANCA, and C‐ANCA was performed. All the tests were negative, and the level of serum complements was normal. Thoracic surgery consults for the dominant cavity in left lung management recommended radiologic intervention by inserting a pneumothorax catheter. Unfortunately, this intervention failed due to technical difficulties. The patient's general condition improved gradually. Cardiac MRI was carried out in another center, and there was no evidence of RA mass or any other significant cardiac abnormalities. Hematuria resolved independently, and we assumed that it was due to trauma during urinary catheterization. As the antifungal treatment continued, the patient's condition improved. We referred the patient for cavity sampling under the CT guide. The sample's PCR test result was positive for aspergillosis. Treatment with intravenous voriconazole continued for 3 weeks in the hospital, and a follow‐up chest CT scan revealed significant improvement (Figure 3). A neurologic consult recommended extending the antiplatelet therapy for 3 months after discharge and performing cardiac Holter monitoring and checking coagulation profile, including fibrinogen level, protein C, protein S, and Antithrombin III level. All of these tests were normal. The Holter monitor device did not reveal any abnormality. The patient was followed for 3 months in the clinic while continuing oral voriconazole. After 3 months, lung cavitary lung lesions disappeared completely, and the patient got symptoms free. The patient's neurologic symptoms also improved, and antiplatelet therapy was terminated after consultation with a neurologist.

## 3 DISCUSSION

The COVID‐19 disease caused by the coronavirus has a broad spectrum of features, in the severe form causing acute respiratory distress syndrome (ARDS).^1^ The previous influenza pandemic described co‐infections and superinfections of viral, bacterial, and fungal infections.^13^ In the current pandemic of COVID‐19, in the studies from China,^7^, ^8^ aspergillosis was isolated from the patient's respiratory tract. Also, in case series from other regions in the world, there were reports of CAPA in about 20%–35% of mechanically ventilated patients.^9^, ^10^, ^11^, ^12^ The median prevalence of CAPA was 9.3% (range from 0% to 33%), and although case definitions were based on different criteria in the studies, CAPA was proven in just four instances, with the remainder having a possible or presumptive diagnosis.^14^

Since there are similarities between influenza and COVID‐19 clinical features, we expect a similar rate of aspergillus infection in COVID‐19 patients.

Nevertheless, aspergillus infection in COVID‐19 patients is uncommon.^15^ In these patients, aspergillosis infection was mainly associated with tracheobronchitis, prior lung disease, prolonged mechanical ventilation, and high immunosuppressor doses or diabetes.^15^ The corticosteroid uses in treating COVID‐19 disease may increase the risk of aspergillus infection. The diagnosis of invasive pulmonary aspergillosis (IPA), according to the European Organization for Research and Treatment of Cancer/Mycosis Study Group (EORTC/MSG), is categorized into proven, probable, and possible. When a combination of host characteristics, clinical manifestations, and positive mycology leads to proven invasive aspergillosis.

Moreover, possible IPA is considered when there are clinical features and host factors but negative mycology results.^16^ The modified definition of invasive pulmonary aspergillosis (AspICU criteria) developed lately and is based on positive results of BAL culture, positive BAL, or serum galactomannan in the absence of histopathology. The radiologic criteria include any pulmonary infiltration rather than EORT radiologic criteria (e.g., halo sign or air‐crescent sign) because this criterion is applied more for neutropenic patients.^17^

European Confederation for Medical Mycology (ECMM) and the International Society for Human Animal Mycology (ISHAM) published a consensus on CAPA definition as proven probable, and possible on 2020, the proven pulmonary case definition was based on invasive growth of aspergillus species in sterile tissues, or positive culture or PCR in the lung biopsy specimen. Probable CAPA is defined as pulmonary radiologic findings of infiltration, nodule or cavitation with mycologic evidence and possible cases as radiologic findings mentioned before and mycologic evidence obtained via non‐bronchoscopic bronchial lavage (NBL).^18^

Chen et al. indicated that the immunocompetent patients with IPA who were hospitalized with influenza showed a significant correlation between using corticosteroids before diagnosis and acquiring IPA.^19^ In the study evaluating reported cases of CAPA in the ICU, steroid exposure, diabetes, and chronic lung disease were the more frequent risk factors.

Moreover, pulmonary nodules and cavitary/halo‐sign were the dominant radiologic findings of the IPA patient,^20^ and cerebral radiological lesions in adult critically ill patients has been reported before by Spapen et al.^21^ Long‐term steroid treatment (16 mg/day or higher prednisone for at least 15 days) was considered as underlying immunosuppression condition in patients with CAPA.^22^ CNS aspergillosis may occur in immunocompetent patients and have a different clinical course and prognosis than immunocompromised patients.^23^ Hematogenous spread of aspergillus can involve all parts of the CNS, including the anterior and middle branches of the cerebral artery, which can be infiltrated by the hyphae leading to direct and indirect damage, especially in frontal, temporal, and parietal lobes.^24^ In a study evaluating CNS aspergillosis in immunocompetent patients, the frequent etiologies of CNS involvement were nasosinusitis, diabetes, and craniotomy. Cerebral infarction occurred in 26.1% of the patients, with meningeal participation.^25^ In this report, we present a patient admitted to our hospital for a neurologic complaint of hemiparesis, without any known underlying disease, including immunodeficiency, except for recent glucocorticoid consumption for COVID‐19 disease treatment for 5 days. We assumed that the neurologic involvement was due to the patient's pulmonary aspergillosis infection. Furthermore, the early starting of the treatment in response to our suspicion of IPA may improve this patient's outcome compared with similar case studies discussed in this paper.^19^, ^25^

## 4 CONCLUSION

The reported cases of CAPA were more in the critically ill patients admitted to the ICU. It is a fascinating feature of aspergillosis infection in a relatively immunocompetent patient with moderate COVID‐19 pneumonia (as a proven case based on positive PCR in lung biopsy specimen), and this may warrant more precaution in prescribing glucocorticoids and other immunosuppressive drugs in order to prevent patients from getting superinfections with these opportunistic organisms and their debilitating complications. Finally, it is essential to consider the possibility of superinfection by these organisms and the other opportunistic infections resulting in starting the appropriate diagnostic evaluations and treatments as soon as possible.

## AUTHOR CONTRIBUTIONS

M Albaji collected the required data of the patient and write the first draft of the manuscript. M R Fattahi has supervised and edited the final draft of the manuscript. A Iranmehr has edited the first draft of the manuscript and consultation for neurological issues of the patients.

## CONFLICT OF INTEREST

The authors declare that they have no conflict of interest.

## ETHICAL APPROVAL

None.

## CONSENT

Written informed consent was obtained from the patient to publish this report in accordance with the journal's patient consent policy.

## Data Availability

Data sharing not applicable to this article as no datasets were generated or analysed during the current study.
